# Estimate of the direct and indirect annual cost of bacterial conjunctivitis in the United States

**DOI:** 10.1186/1471-2415-9-13

**Published:** 2009-11-25

**Authors:** Andrew F Smith, Curtis Waycaster

**Affiliations:** 1 Medmetrics Inc, 30 Charles Street, Ottawa, Ontario, K1 M 1R2, Canada; 2 Adjunct Professor (Health Economics), McGill University, Suite 102, 3550 University Street, Montreal, Quebec, H3A 2A7, Canada; 3 Health Economics, Alcon Laboratories, TC-41, 6201 South Freeway, Fort-Worth, Texas, 76143-2099, USA

## Abstract

**Background:**

The aim of this study was to estimate both the direct and indirect annual costs of treating bacterial conjunctivitis (BC) in the United States. This was a cost of illness study performed from a U.S. healthcare payer perspective.

**Methods:**

A comprehensive review of the medical literature was supplemented by data on the annual incidence of BC which was obtained from an analysis of the National Ambulatory Medical Care Survey (NAMCS) database for the year 2005. Cost estimates for medical visits and laboratory or diagnostic tests were derived from published Medicare CPT fee codes. The cost of prescription drugs was obtained from standard reference sources. Indirect costs were calculated as those due to lost productivity. Due to the acute nature of BC, no cost discounting was performed. All costs are expressed in 2007 U.S. dollars.

**Results:**

The number of BC cases in the U.S. for 2005 was estimated at approximately 4 million yielding an estimated annual incidence rate of 135 per 10,000. Base-case analysis estimated the total direct and indirect cost of treating patients with BC in the United States at $ 589 million. One- way sensitivity analysis, assuming either a 20% variation in the annual incidence of BC or treatment costs, generated a cost range of $ 469 million to $ 705 million. Two-way sensitivity analysis, assuming a 20% variation in both the annual incidence of BC and treatment costs occurring simultaneously, resulted in an estimated cost range of $ 377 million to $ 857 million.

**Conclusion:**

The economic burden posed by BC is significant. The findings may prove useful to decision makers regarding the allocation of healthcare resources necessary to address the economic burden of BC in the United States.

## Background

### Natural history

Bacterial conjunctivitis is a microbial infection of the mucous membrane of the conjunctiva of the eye and can occur in both adults and children. It is produced by an array of microorganisms, with the most common bacterial pathogenic organism worldwide being *Staphylococcus aureus *[1-4]. Other prominent bacterial species include: *Streptococcus pneumoniae *and *Haemophilus influenzae*, while other *Staphyloccocus *species, *Moraxella *species and opportunistic bacteria are more typically seen in the chronic forms of bacterial conjunctivitis [1-4]. *Haemophilus influenzae *is the most common isolate in children less than seven years of age [4]. In persons with symptoms of conjunctivitis, who are sexually active, they may be infected with either gonorrhea or chlamydia. In most instances, the infection begins unilaterally, with the fellow eye becoming involved within a few days. Although, it is typically considered a minor infection, bacterial conjunctivitis can have a considerable impact on school attendance, lost work time and very occasionally, can result in permanent or sight-threatening sequelae such as bacterial keratitis and endophthalmitis in extreme cases [5,6].

### Epidemiology

Although the literature on the epidemiology of bacterial conjunctivitis contains several references to its highly contagious nature [7-10] no overall population-based data exists on the incidence of bacterial conjunctivitis. In the United States it is estimated that 23% of bacterial conjunctivitis cases occur in the 0-2 year age range, 28% occur in the 3-9 year range, 13% occur in the 10-19 year range with the remaining 36% of cases occurring in adults [11,12].

Existing clinic-based estimates from Norway, have calculated that the prevalence of the most severe form of acute infective bacterial conjunctivitis is on the order of 30 out of 1000 patients in a general medical practice, though a correct diagnosis was only made in about two-thirds of these cases [13]. Similar clinic-based data from the United Kingdom have pointed to a rise in the proportion of patients who have sought medical attention for conjunctivitis, rising from 284 per 10,000 in 1981-1982 to 395 per 10,000 over the period 1991-1992 [14]. It has further been estimated that acute bacterial conjunctivitis represents up to 1% of all visits to general practitioners in the United Kingdom [15]. However, it has also been noted that general practitioners tend to over-diagnosis bacterial conjunctivitis [15] due to the difficulties of differentiating between the bacterial and viral forms of the disease. In 2002 a survey of general practitioners in the United Kingdom found that only 36% felt that they could correctly differentiate between acute bacterial and viral conjunctivitis. Additionally, 95% of the physicians surveyed indicated they prescribed topical antibiotics to patients with suspected bacterial conjunctivitis [16].

### Existing Economic Evidence

The treatment and management of U.S. bacterial conjunctivitis patients has never been fully quantified and the costs incurred by the healthcare system remain largely unknown. Several studies found that in both the Netherlands and the United Kingdom, 900,000 and 3.4 million prescriptions, respectively, for topical ocular antibiotics were issued at a cost of £5.9 million and £4.7 million, respectively. However, these studies included all aetiologies which gave rise to a prescription for topic ocular antibiotics and were not necessarily due to bacterial conjunctivitis [17-19]. It is against this background, that the current study attempts to estimate the annual cost of healthcare resource utilization attributable to the management of bacterial conjunctivitis in the United States.

### Cost of Illness Studies

The economic impact of a given disease is commonly measured as an aggregate of both the direct and indirect costs associated with treating and managing patients with the given condition. A third category of "intangible costs" or costs due to pain and suffering are sometimes included in the overall panoply of costs. However, in practice intangible costs are difficult to quantify and are typically not considered. Direct costs are defined as the value of goods and services used in the treatment, care, and rehabilitation of a given illness or injury [20-23]. Indirect costs are defined as the value of economic output lost because of illness, injury-related work disability, or premature death [20-23]. Additionally, indirect costs include the value of time lost from work and leisure activities by family members or friends who have forgone these activities when providing care for patients.

The objective of this research was to estimate the total cost of bacterial conjunctivitis in the United States. Both direct and indirect costs were estimated from the societal or overall healthcare system perspective.

## Methods

### Study Design

This study used an incidence-based burden-of-illness framework to estimate the annual costs of bacterial conjunctivitis in the United States. A bottom-up costing approach was used to estimate the average direct and indirect costs for the typical treatment and management of a patient with bacterial conjunctivitis. These average costs were then multiplied by the estimated annual incidence rate to determine the total annual cost. Since a typical episode of bacterial conjunctivitis is approximately 10-14 days in duration no discounting was performed and all costs are presented in 2007 US dollars.

### Data Sources

A comprehensive literature search was performed using Medline^©^, Embase^©^, Health Star^©^, NHSEED^©^, Cochrane^© ^and CINAHL^® ^databases for articles on bacterial conjunctivitis. The search strategy focused on articles related to the clinical management of patients with bacterial conjunctivitis, its epidemiology and costs associated with its treatment. The keywords used to populate the literature search included: bacterial conjunctivitis, conjunctivitis, treatment and management, epidemiology, incidence, prevalence, health care costs and resource utilization. The search was restricted to English language articles published within the last twenty years. In addition, a manual literature search was also performed on citations in the published articles to identify any relevant articles missed in the initial search. Studies providing evidence of the incidence of bacterial conjunctivitis were also used to inform our analysis.

### Physician Costs

Data on the office cost associated with visiting either a pediatrician, general practitioner or ophthalmologist due to bacterial conjunctivitis were derived from an examination of the Healthcare Common Procedure Coding System (HCPCS Codes) commonly used by the Center for Medicare and Medicaid Services. These codes are based on the Current Procedural Technology (CPT) codes developed by the American Medical Association to monitor fees for various medical procedures. According to the Center for Medicare and Medicaid Services, part of the U.S. Department of Health and Human Services, the key CPT codes of interest are: i) 99213 defined as "An office other outpatient visit for the evaluation and management of an established patient ... Usually, the presenting problem(s) are of low to moderate severity. Physicians typically spend 15 minutes face-to-face with the patient and/or family" and ii) 92004 defined as "(Ophthalmological services) Medical examination and evaluation with initiation of diagnostic treatment program; comprehensive, new patient, one or more visits" [24]. Data on the mean values associated with each of these CPT fee codes for 2005 are presented in Table 1. The use of the Medicare CPT fee codes was selected as it was most likely to approximate the cost of seeing either a pediatrican, general physician or ophthalmologist across the United States.

**Table 1:** Primary Current Procedural Technology (CPT) codes used by Medicare and Medicaid Services mean values for 2005 *

CPT Code	Mean Monetary Value (US $)
Outpatient visit physician examination **99213**	53.50

Ophthalmological services initial visit **92004**	131.35

Combination of values for **99213 **and **92004**	92.43

* Values were derived from an examination of the HCPC codes used by the Centre for Medicare and Medicaid Services (CMS) in the United States for the year 2005

### Incidence of Bacterial Conjunctivitis

Data from the 2005 National Ambulatory Medical Care Survey (NAMCS) were used to estimate the incidence rate of bacterial conjunctivitis in the United States [25]. The NAMCS is a national sample survey designed to meet the need for objective, reliable information about the provision and use of ambulatory medical care services in the United States. Data were obtained on patients' symptoms, physicians' diagnoses, and medications ordered or provided. The survey also provides statistics on the demographic characteristics of patients and services provided, including information on diagnostic procedures, patient management, planned future treatment, geographic location, racial composition and the extent of insurance coverage.

Data from the NAMCS dataset were extracted using the International Classification of Diseases 9th edition (ICD-9) codes according to the following criteria. A case of bacterial conjunctivitis was considered to obtain at least one of the following three ICD-9 fields: namely, 372.30 (Conjunctivitis, unspecified), 372.03 (Conjunctivitis, mucopurulent) or 372.00 (Acute conjunctivitis) [26]. This choice of ICD-9 codes enabled the broadest possible definition of a case of BC to be detected in the NAMCS database.

### Other Direct Costs

Resource utilization and direct healthcare costs were divided into three main categories; 1) Medical visit costs, 2) costs due to diagnostic tests and 3) the cost of prescription drugs. Resource costs were calculated in US dollars at 2007 prices. The costs of diagnostic tests, such as bacterial cultures, were also obtained from an examination of the CMS data set using the relevant CPT fee code for conducting a bacterial culture, namely, CPT code number 87070, while data on the medications prescribed for bacterial conjunctivitis were obtained from the 2005 NAMCS dataset [27]. Price data for ophthalmic antibiotics identified in the NAMCS dataset were obtained from a standard reference for drug pricing, the Red Book (2004 Edition) as this reflected prices at the start of 2005, the year which was analysed in the NAMCS dataset [28].

### Indirect Costs

In order to estimate indirect costs, it was assumed that for each child's visit to the doctor's office, an adult accompanied their child, resulting in lost time from work. By the same token, adults seeking treatment for bacterial conjunctivitis also lost earnings due to lost time from work. It was assumed that a medical visit resulted in approximately two hours of lost productivity for adult patients or adult caregivers of children seeking medical attention for bacterial conjunctivitis. The U.S. Bureau of Labor Statistics estimated the 2005 median weekly wage of all US workers across all industries at $651 USD per week [29]. Using this figure, the average hourly wage rate in the United States for a forty hour work week equals $16.28 per hour USD. In addition, one must take into account the fraction of persons who were unemployed. Again according to the U.S. Bureau of Labor Statistics during 2005, 63% of workers were employed while 37% of were unemployed and or retired [29]. For that fraction of the population which was unemployed, a minimum wage rate of $ 5.15 per hour was used to value the foregone earnings potential had these persons been in paid employment [29]. Using these figures, the indirect cost due to lost productivity was estimated using the following formula ($ 16.28 × 0.63) + ($ 5.15 × 0.37) = $10.26 + $ 1.91 = $ 12.17 per hour, or $ 24.34 for the two hour period per patient per medical visit due to bacterial conjunctivitis.

### Economic Model

In order to estimate the total costs of bacterial conjunctivitis (BC) in the United States, the following simplified economic model was developed. Thus the total cost was found by adding up both the total direct and total indirect costs according to the following formula:

where;

α = Number of cases of BC seen by pediatricians as calculated using the NAMCS database;

β = Direct cost associated with a pediatric visit (mean physician visit cost + mean cost for lab tests + mean cost of drug therapy)

γ = Indirect costs associated with a pediatric visit

δ = Number of cases of BC seen by all other physicians in NAMCS database (Total BC Visits - Pediatrician visits recorded in the NAMCS dataset)

λ = Direct cost associated with a visit to all other physicians combined (mean of general physician visit cost plus mean ophthalmologists visit cost) + mean cost for lab tests + mean cost of drug therapy)

η = Indirect cost associated with a visit to all other physician visits combined.

### Sensitivity Analysis

A deterministic sensitivity analysis approach recommended by Briggs and Gray was conducted to test the robustness of the economic model [30]. Both one-way and two-way sensitivity analyses on the annual incidence of bacterial conjunctivitis and treatment costs were performed using a range of ± 20%. This range allowed sufficient variation in both incidence rates and cost figures to be explored in relation to the overall values obtained in the base-case analysis.

## Results

### Incidence of Bacterial Conjunctivitis

Using the three ICD-9 codes of 372.30 (Conjunctivitis, unspecified), 372.03 (Conjunctivitis, mucopurulent) or 372.00 (Acute conjunctivitis), the 2005 NAMCS data set showed an estimated 4,016,544 visits to ambulatory physicians for bacterial conjunctivitis were made. Of these visits, 2,270,268 (56.5%) were made by females and 1,746,276 (43.5%) by males [25]. Furthermore, 1,356,693 (33.77%) visits were made by patients less than 15 years of age to pediatricians [25]. It was assumed that each visit represented a unique patient and there were no repeat visits by the patient during the year. Thus, using a 2005 US base population of 296,507,061 the crude incidence rate for bacterial conjunctivitis was estimated at 4,016,544/296,507,061 (1.3%) or roughly 135 cases per 10,000 annually [31].

### Geographic, Racial and Insurance Coverage within the NAMCS dataset

Table 2 provides a distribution of key geographic, racial and health insurance coverage variables for bacterial conjunctivitis ambulatory physician visits within the 2005 NAMCS data set. The majority of 2005 bacterial conjunctivitis cases (31%) occurred in the Southern region of the U.S. followed by the Midwest (28%), West (23%) and Northeast (18%) regions. With respect to the racial distribution of bacterial conjunctivitis cases, the vast majority of patients were listed as White (84%) followed by Asian (10%), Black (5%) with the remainder (1%) listed as being of other racial descent. The majority of bacterial conjunctivitis cases in the 2005 NAMCS data set indicated that they were covered by private insurance (62%) followed by Medicaid/SCHIP (18%), Medicare (11%), with the remainder listed as other.

**Table 2:** Distribution of key Geographic, Racial and Health Insurance Coverage Variables in the NAMCS data set 2005

VARIABLE OF INTEREST	NUMBER (N = 4,016,544)	PERCENTAGE (%)
**Geographic Location**		

South	1,250,438	31.13%

Midwest	1,129,668	28.13%

West	912,634	22.72%

Northeast	723,804	18.02%

**Racial Distribution**		

White	3,391,138	84.43%

Asian	384,943	9.58%

Black	188,948	4.70%

Other	51,515	1.29%

**Insurance Coverage**		

Private insurance	2,484,655	61.86%

Medicaid/SCHIP*	718,271	17.88%

Medicare	451,384	11.24%

Other	362,234	9.02%

* State Children's Insurance Health Program

It is interesting to note that the relative proportions of bacterial conjunctivitis cases in the NAMCS dataset is representative of the geographic regions of the United States. However, there appears to be some disparity in terms of the racial mix of bacterial conjunctivitis cases. US Census figures for the period 2006-2008 show that the acutal percentage population distribution of White Americans is 74.3%, while for Black Americans it is 12.3% of the total population [32]. Equally interesting is that the percentage of private insured patients captured in the NAMCS dataset, i.e., at 51%. This is higher than the US national average of about 36% [33].

### Prescribed Ophthalmic Antibiotic Drug Costs

The relative proportions of prescribed drugs for bacterial conjunctivitis were estimated from the 2005 NAMCS data set. Table 3 provides the most frequently prescribed ophthalmic antibiotics within the NAMCS data set. Using this information, the costs of prescribed medications used to treat bacterial conjunctivitis were determined from the average wholesale prices listed in the 2004 Red Book[28]. Drug cost data are presented in Table 3 and form the basis used to calculate the overall mean cost of prescribed medications used to treat persons with bacterial conjunctivitis.

**Table 3:** Cost of Prescribed Ophthalmic Antibiotics

PRESCRIBED OPHTHALMIC ANTIBIOTIC	PERCENTAGE (%) DISTRIBUTION WITHIN THE NAMCS DATASET	AVERAGE WHOLESALE PRICE (RED BOOK 2004)
Sulfacetamide sodium 10%, 15 ml	19%	$2.30

Moxifloxacin 0.5%, 3 ml	16%	$46.80

Polymyxin/Trimethoprim 10,000 U/1 mg/ml, 10 ml	14%	$17.42

Tobramycin 0.3%, 5 ml	14%	$49.08

Tobramycin/Dexamethasone 0.3%- 0.1%, 5 ml	12%	$59.88

Gentamicin 0.3%, 5 ml	11%	$3.95

Ciprofloxacin 0.3%, 5 ml	6%	$79.38

Neomycin/Polymyxin/Bacitracin 10,000 U/3.5 mg/400 U/g, 3.5 g	4%	$15.77

Bacitracin 500 U/g, 3.5 g	3%	$15.77

Sulfacetamide 10%, 3.5 g	1%	$8.10

Levofloxacin 0.5%, 5 ml	1%	$21.91

**Weighted Average Price (US $)**		**US $ 31.02**

### Direct and Indirect Costs

Table 4 provides the key unit costs used in the economic model for the base-case and sensitivity analyses. These cost data were obtained from a review of the CPT fee codes for the costs associated with laboratory cultures for bacterial conjunctivitis and used a CPT code of 87070 and the indirect cost for lost productivity. As previously noted, indirect costs were limited to lost wages due to time spent seeking medical attention for the treatment of bacterial conjunctivitis.

**Table 4:** Other Direct and Indirect Costs (Figures in US$)

RESOURCE CATEGORY	MEAN COST PER VISIT (US $)
**Direct Costs**	

Labs Tests for Cultures (CPT fee code = 87070)*	$12.0375

**Indirect Costs**	

Indirect Costs (Lost productivity of approximately two hours per visit)	$24.34

*The mean cost for lab tests was obtained by surveying the CPT fee codes contained in the CMS database.

### Base-Case Scenario

As can be seen from Table 5, the base-case scenario produced an estimated direct and indirect cost of treating patients with bacterial conjunctivitis in the United States of $ 491 million and $

97 million, respectively, for a total cost of $ 589 million. The base case scenario assumes there is no variation in either the annual incidence of bacterial conjunctivitis or costs associated with the treatment and management of patients with this condition.

**Table 5:** Base-case analysis

VARIABLE OF INTEREST	NUMBER
Total annual incidence of bacterial conjunctivitis(visits)	4,016,544

Total annual incidence of bacterial conjunctivitis treated by pediatricians(visits)	1,356,693

Total annual incidence of bacterial conjunctivitis treated by all other physicians(visits)	2,659,851

Total annual direct cost of bacterial conjunctivitis treated by pediatricians(US$)	$130,988,709

Total annual indirect cost of bacterial conjunctivitis treated by pediatricians(US$)	$33,021,908

Total annual direct cost of bacterial conjunctivitis treated by all other physicians(US$)	$360,356,613

Total annual indirect cost of bacterial conjunctivitis treated by all other physicians(US$)	$64,740,773

Total annual direct cost of bacterial conjunctivitis treated by all medical personnel (pediatrician's plus all other physicians) (US$)	$491,345,323

Total annual indirect cost of bacterial conjunctivitis treated by all medical personnel (pediatrician's plus all other physicians) (US$)	$97,762,681

Total annual direct and indirect cost of bacterial conjunctivitis treated by all medical personnel (pediatrician's plus all other physicians) (US$)	$589,108,004

### One-way sensitivity analysis

Table 6 presents the results of a one-way sensitivity analysis assuming a 20% variation in the annual incidence of bacterial conjunctivitis while holding treatment costs constant. This generates a cost range of $ 469 million to $ 705 million. As with the base-case results, this figure is an aggregate of the cost of treating both pediatric and adult patients. Additionally, a two-way sensitivity analysis was performed wherein the annual incidence of bacterial conjunctivitis and the costs of treating and managing patients with bacterial conjunctivitis were varied simultaneously.

**Table 6:** One-way sensitivity analysis varying Incidence of Bacterial Conjunctivitis

VARIABLE OF INTEREST	20% INCREASE IN INCIDENCE OF BC	20% DECREASE IN INCIDENCE OF BC
Total annual incidence of bacterial conjunctivitis (visits)	4,819,853	3,213,235

Total annual incidence of bacterial conjunctivitis treated by pediatricians (visits)	1,628,032	1,085,354

Total annual incidence of bacterial conjunctivitis treated by all other physicians (visits)	3,191,821	2,127,881

Total annual direct cost of bacterial conjunctivitis treated by pediatricians (US$)	$157,186,490	$104,790,929

Total annual indirect cost of bacterial conjunctivitis treated by pediatricians (US$)	$37,998,267	$26,417,516

Total annual direct cost of bacterial conjunctivitis treated by all other physicians (US$)	$432,427,909	$288,285,318

Total annual indirect cost of bacterial conjunctivitis treated by all other physicians (US$)	$77,688,923	$49,664,743

Total annual direct cost of bacterial conjunctivitis treated by all medical personnel (pediatrician's plus all other physicians) (US$)	$589,614,399	$393,076,247

Total annual indirect cost of bacterial conjunctivitis treated by all medical personnel (pediatrician's plus all other physicians) (US$)	$115,687,190	$76,082,259

Total annual direct and indirect cost of bacterial conjunctivitis treated by all medical personnel (pediatrician's plus all other physicians) (US$)	$705,301,589	$469,158,505

### Two-way sensitivity analysis

Table 7 presents the results of a simultaneous two-way sensitivity analysis on both the annual incidence and costs of treating and managing patients with bacterial conjunctivitis. Assuming a 20% variation in both the annual incidence of bacterial conjunctivitis and treatment costs occurring simultaneously, the result is an estimated cost range of $ 377 million to $ 857 million.

**Table 7:** Two-way sensitivity analysis varying both incidence and cost of treatment and management of patients with bacterial conjunctivitis

VARIABLE OF INTEREST	20% INCREASE IN INCIDENCE OF BC PLUS 20% INCREASE IN COSTS OF TREATING AND MANAGING PATIENTS WITH BC	20% DECREASE IN INCIDENCE OF BC PLUS 20% DECREASE IN COSTS OF TREATING AND MANAGING PATIENTS WITH BC
Total annual incidence of bacterial conjunctivitis (visits)	4,819,853	3,213,235

Total annual incidence of bacterial conjunctivitis treated by pediatricians (visits)	1,628,032	1,085,354

Total annual incidence of bacterial conjunctivitis treated by all other physicians (visits)	3,191,821	2,127,881

Total annual direct cost of bacterial conjunctivitis treated by pediatricians (US$)	$188,623,788	$83,832,743

Total annual indirect cost of bacterial conjunctivitis treated by pediatricians (US$)	$47,554,815	$21,131,842

Total annual direct cost of bacterial conjunctivitis treated by all other physicians (US$)	$528,501,721	$230,628,254

Total annual indirect cost of bacterial conjunctivitis treated by all other physicians (US$)	$93,233,091	$41,429,843

Total annual direct cost of bacterial conjunctivitis treated by all medical personnel (pediatrician's plus all other physicians) (US$)	$717,125,509	$314,460,997

Total annual indirect cost of bacterial conjunctivitis treated by all medical personnel (pediatrician's plus all other physicians) (US$)	$140,787,906	$62,561,685

Total annual direct and indirect cost of bacterial conjunctivitis treated by all medical personnel (pediatrician's plus all other physicians) (US$)	$857,913,415	$377,022,683

Both the one and two-way sensitivity analyses demonstrate that given the economic model used, variations in the incidence and in the unit costs have relatively large effects on the overall total costs associated with treating bacterial conjunctivitis in the United States. As such, our model is sensitive to changes in both incidence and unit cost data used to populate the economic model.

## Discussion

In terms of situating the findings of the current study within the context of other bacterial conjunctivitis epidemiology studies, it is worth noting that NAMCS is a national survey which was originally designed to meet the need for objective, reliable information about the provision and use of ambulatory medical care services in the United States. This said, the NAMCS survey was designed to track medical conditions and prescribing patterns and provides reliable estimates on the patient-physician encounter. Using a refined ICD-9 classification for extracting cases of bacterial conjunctivitis, it was possible to estimate an annual bacterial conjunctivitis incidence rate of 135 per 10,000 in the United States. This U.S. bacterial conjunctivitis incidence rate is less than one-half of the rates reported in Norway (30 per 1,000) and the United Kingdom (284 per 10,000) [13,14]. It is interesting to note that even using a case definition of BC as any one of the three ICD-9 codes (372.30 Conjunctivitis, unspecified), 372.03 (Conjunctivitis, mucopurulent) or 372.00 (Acute conjunctivitis) our analysis produced a significantly lower incidence rate of BC in the US versus those obtained in previous studies.

Our research raises questions as to why there is such a difference between the U.S. and European bacterial conjunctivitis incidence rates. One possible source of disparity may be due to study methodologies. In the European studies the incidence rates were derived from actual clinical data while the NAMCS figure was derived from a physician-patient encounter database using ICD-9 disease classifications. A second source of distinction in incidence rates may be due to history. The higher incidence rates in the UK and Norway were reported in 1981 and 1991, respectively. This represents a time span of 24 years from the earliest study to this study which uses 2005 data. Since 1991 many new antibiotics have been marketed for the treatment of bacterial conjunctivitis in the United States. However, according to Hovding topical chloramphenicol is still the mainstay of bacterial conjunctivitis therapy in the United Kingdom and Europe [13].

Another factor which may explain the divergence in bacterial conjunctivitis incidence rates between Europe and the U.S. is the medical practice patterns and physician's attitudes towards the treatment of bacterial conjunctivitis. American doctors may be more aggressive in treating bacterial conjunctivitis with antibiotics than their European counterparts. The effects of delayed antibacterial treatment may influence the transmission rate of bacterial conjunctivitis and consequently affect the incidence rates.

From a health economics perspective, it is possible to compare the costs obtained in the current paper and to examine them in relation to other cost of illness studies which have been conducted for vision related disorders in the United States and elsewhere during roughly the same time period. Thus, assuming that the annual cost of treating and managing patients with bacterial conjunctivitis is as given in the base-case analysis, i.e., $ 589 million, it is possible to contrast this against other cost of illness studies in order to better understand its relative impact.

A review of cost of illness studies conducted in eye diseases in 2004, found that the total financial cost of major visual disorders among US residents over 40 years of age was $ 35.4 billion, with $ 16.2 billion due to direct medical costs, $ 11.1 billion due to other direct costs and $ 8 billion due to lost productivity [34]. The authors further calculated that the direct medical costs for selected blinding conditions in the United States amounted to: $ 6.8 billion for cataracts, $ 5.5 billion for refractive errors, $ 2.9 billion for glaucoma, $ 575 million for age related macular degeneration and $ 493 million for diabetic retinopathy [34]. Similarly, in 2006, for example, Vitale *et al *estimated that the annual direct cost of correcting distance vision impairment in the United States was $ 3.8 billion [35]. It has also been estimated by Frick *et al *that as much as $ 5.5 billion is spent annually on the direct medical care of blind individuals in the United States [36].

For international comparison purposes, a recent report on the economic burden of bilateral neovascular age-related macular degeneration in five countries, namely Canada, France, Germany, Spain and the UK found that the annual societal cost due blindness ranged from 268 to 1311 million Euros [37]. Equally, Happich *et al *found that the economic burden of diabetic retinopathy in Germany in 2002 was 3.51 billion Euros if measured from the societal perspective [38]. Thus when viewed against the above studies, the cost of illness due to bacterial conjunctivitis is on a par with other more chronic vision problems, such as ARMD and diabetic retinopathy which affect relatively small numbers of individuals on an annual basis but accrue greater per patient costs. Bacterial conjunctivitis, by contrast, accrues relatively smaller per patient costs, yet it affects a far greater number of persons on an annual basis than does ARMD or diabetic retinopathy.

Moreover, in comparing the bacterial conjunctivitis cost findings with the above mentioned cost of illness studies in vision and eye disease, it is important to remember that our study sought to conservatively measure both the direct and indirect costs associated with seeking treatment due to bacterial conjunctivitis. Further refinement of both the direct and indirect costs data would undoubtedly increase the overall total costs attributable to bacterial conjunctivitis. The current analysis, for example, used a highly conservative estimate of the indirect costs associated with lost productivity due to seeking medical attention for the treatment of bacterial conjunctivitis, taking into account only lost wages and did not include other indirect costs such as travel, transportation and parking costs which were difficult to fully quantify with any degree of precision. Our findings must therefore be regarded as a highly conservative estimate of the annual costs due to the treatment and management of patients with bacterial conjunctivitis in the United States.

### Future Study

One area for future study would be to investigate if the introduction of newer more potent antibiotics has exerted any influence over bacterial conjunctivitis incidence rates. There is evidence that the fourth generation fluoroquinolone, and moxifloxacin, exhibited better efficacy compared to polymyxin/trimethoprim, an older combination antibiotic agent [39]. Cost-effectiveness evaluations of this question, looking at total costs, would be useful as would those studies which attempt to model the indirect costs associated with microbial drug resistance, though this topic is far beyond the scope the present analysis.

## Conclusion

The burden of bacterial conjunctivitis is considerable both in absolute terms, i.e., is, the number of patients treated on an annual basis and in economic terms, i.e., the direct costs of physician's visits, drug therapy, diagnostic tests and the indirect costs of lost earnings. Infectious diseases pose a unique set of problems relating to the burden of illness studies because the transmission rate of the illness to susceptible persons directly influences the incidence rate of the disease. Since costs are sensitive to the incidence rate, anything that affects the contagious period of the infected population can influence the incidence rate and consequently the cost-of-illness. The use of potent antibiotics may reduce the contagious period of the infected population thereby reducing the transmission of the disease to the susceptible population. Consequently, there may well be a potential role for use of potent antibiotics, such as the newer fourth-generation fluoroquinolones to reduce the aggregate cost of bacterial conjunctivitis, especially those costs related to indirect costs, even if the direct drug costs may be higher in the first instance.

## Competing interests

This study was sponsored financially by Alcon Laboratories Ltd. Dr Curtis Waycaster is an employee of Alcon Laboratories, Ltd. Dr Andrew F. Smith was previously employed by Alcon Laboratories Ltd. However, the authors have no other potential conflicts of interest that are directly relevant to the content of this manuscript.

## Authors' contributions

**AFS:** Data set acquisition, data analysis plan, preliminary and principal data analysis, design of health economic model, collection of health economic cost data, manuscript preparation and revision.

**CW: **Assistance with data analysis plan, input with health economic model and results section, significant manuscript preparation and revision.

## Authors' informations

**Andrew F. Smith, PhD **is graduate of McGill University, Montreal, Canada and the Faculty of Medicine, University of London, England. Dr Smith is an Adjunct Professor (Health Economics) at McGill University. From 2001 to 2007, Dr Smith was Regional Health Economics Manager for Europe, Mediterranean and Africa for Alcon Laboratories Ltd. In 2007 Dr Smith founded Medmetrics Inc., a health economics research and consulting company specializing in all aspects of the design, analysis, reporting and publication of health economic studies.

**Curtis Waycaster, PhD **has been a Senior Outcomes Researcher in the Health Economics Department of Alcon Laboratories Ltd, since 2001. Dr Waycaster is responsible for a wide range of both medical device and pharmaceutical products manufactured by Alcon Laboratories Ltd.

## Pre-publication history

The pre-publication history for this paper can be accessed here:

http://www.biomedcentral.com/1471-2415/9/13/prepub
